# Nutritional Supplementation to Preserve Healthy Lean Mass and Function During Periods of Pharmacological and Nonpharmacological‐Induced Weight Loss: Protocol for a Systematic Review and Meta‐Analysis

**DOI:** 10.1002/hsr2.70219

**Published:** 2024-12-05

**Authors:** Emily James, Ryan J. Kelsey, Jack A. Sargeant, Joe Henson, Tom Yates, Thomas J. Wilkinson

**Affiliations:** ^1^ Diabetes Research Centre University of Leicester Leicester UK; ^2^ NIHR Leicester Biomedical Research Centre, Leicester General Hospital Leicester UK; ^3^ Leicester Diabetes Centre University Hospitals of Leicester NHS Trust Leicester UK

**Keywords:** frailty, obesity, sarcopenia, skeletal muscle, weight loss

## Abstract

**Background and Aims:**

Weight loss without adequate anabolic stimuli can lead to reductions in lean, as well as adipose, tissue. Health benefits typically associated with weight loss are therefore attenuated, as body composition potentially shifts from an obese to sarcopenic/frail phenotype. In contrast to the traditional connotation of frailty, this might occur in younger adults and is characterized by reduced performance in functional tasks of daily living. Nutritional supplements as an adjunct to weight loss intervention could support the maintenance of lean mass, particularly if structured exercise is contraindicated. This systematic review aims to quantify the effect of nutritional supplements, without concurrent exercise, on lean mass in individuals undergoing pharmacological and nonpharmacological‐induced weight loss.

**Methods:**

This protocol describes a prospective systematic review and meta‐analysis of randomized controlled trials investigating the effect of nutritional supplements on lean mass during weight loss. Comparison arms will not use nutritional supplements. Literature searches will be conducted using the following online databases: Embase, MEDLINE, Web of Science, Google Scholar, and OpenGrey. Outcome measures related to body composition, lean mass, skeletal muscle mass/size, or physical function will be extracted. Risk of bias will be assessed using the United States National Heart Lung and Blood Institute quality assessment tool for controlled intervention studies. A meta‐analysis will be conducted to synthesize comparable outcomes.

**Results:**

The results of this review will be reported in adherence to PRISMA (Preferred Reporting Items for Systematic Review and Meta‐Analysis Protocols) standards, and published in a peer‐reviewed journal.

**Conclusion:**

This study will be the first to systematically review nutritional interventions for the preservation or accretion of lean mass during weight loss. This review will identify gaps in the literature and inform the development of optimized weight loss strategies, for use in research and practice.

**Trial Registration:**

International Prospective Register of Systematic Reviews: PROSPERO CRD42024521540.

## 1 Introduction

Weight loss is an important goal in the management of, and amelioration of metabolic risk factors for, several chronic conditions, including obesity, Type 2 diabetes mellitus, and cardiovascular disease [1]. Recent advances in pharmacological and nonpharmacological weight loss therapies have led to improvements in metabolic risk profile and substantial weight loss, in those to which they are prescribed [2]. Ideally, weight loss should be predominantly derived from the fat mass compartment (the main driver of metabolic disease). However, the potential health benefits of induced weight loss may be compromised by a concomitant loss of lean mass in the region of ~25% [3, 4, 5, 6]. This phenomenon has been observed as a result of weight loss secondary to pharmacological (e.g., GLP‐1RA therapy) [6], surgical [7], and lifestyle interventions [8]. Any loss of any lean mass may reduce physical function and increase the risk of developing sarcopenia (low skeletal muscle mass and impaired muscle function) and/or frailty [4]. Strategies to preserve skeletal muscle and improve physical function during weight loss should be prioritized. Currently, the addition of structured exercise is the most widely evidenced therapy to preserve lean mass during weight loss [3, 9, 10].

Aside from exercise, novel nutritional interventions may provide an alternative therapeutic approach to maintaining muscle mass, strength, and function during weight loss, particularly for people with contraindications to exercise (including those recovering after surgery, or with other health conditions contraindicating safe exercise or physical activity, such as some people living with Long Covid [11]). Nevertheless, there is a paucity of evidence in this regard. Whilst studies have demonstrated the favorable effects of supplements like vitamin D and b‐hydroxy‐b‐methylbutyrate on muscle mass and strength [3, 12], no study has attempted to systematically review the role of adjunct nutritional supplements on muscle health during induced and substantial weight loss. To optimize the treatment of weight loss and guide future research, nutritional supplements that preserve or improve muscle health first need to be identified and the intervention effect quantified.

This systematic review primarily aims to evaluate the effectiveness of nutritional supplements, without concurrent exercise, in preserving lean mass in individuals undergoing pharmacological and nonpharmacological‐induced weight loss. Secondary outcomes will investigate the intervention effect on other measures of body composition and muscle function/performance/strength.

## 2 Methods

This systematic review protocol is registered on the International Prospective Register of Systematic Reviews (PROSPERO) (Registration number: CRD42024521540). The protocol has been developed in adherence to PRISMA‐P (Preferred Reporting Items for Systematic Review and Meta‐Analysis Protocols) standards [13] (see Supplementary Material S1).

### 2.1 Eligibility Criteria

Studies will be selected according to the criteria outlined in Table 1.

**Table 1:** Inclusion and exclusion criteria.

	Inclusion	Exclusion
Study designs	We will include randomized controlled trials (RCTs), including cluster RCTs and randomized cross‐over studies.	We will exclude non‐RCTs, case series, and case reports.
Participants	We will include studies examining any population undergoing pharmacological and/or nonpharmacological‐induced weight loss, including but not limited to: Diet‐induced weight loss (e.g., ketogenic, low‐calorie diet [LCD]) Weight loss induced as a result of bariatric/weight loss surgery Weight loss induced as a result of pharmacological medication (e.g., SGLT2i, GLP‐1RA)	Studies will be excluded where weight loss has been induced as a result of an exercise/physical activity‐based intervention.
Interventions	Taking a broad perspective, of interest are interventions addressing the following: Adjunct nutritional supplementation (used in addition to the primary means of weight loss) that is primarily designed/being used to improve or preserve body composition, lean mass, skeletal muscle mass/size, or physical function (e.g., chromium picolinate, caffeine, nitrate, protein supplementation, vitamin D, polyunsaturated fatty acids [PUFA], citulline, b‐hydroxy‐b‐methylbutyrate, branched‐chain amino acid, creatine monohydrate, fish‐oil–derived n–3 fatty acid). Any intervention duration and frequency or dose of supplement will be eligible for inclusion.	Studies will be excluded where, in the intervention group, supplementation is *combined with* any additional substantial physical activity intervention (e.g., supervised structured exercise training).
Comparators	Any appropriate comparator group or study arm that *does not include nutritional supplementation* primarily designed/being used to improve or preserve body composition, skeletal muscle mass, or physical function.	
Outcomes	The primary outcome of interest is lean mass using any assessment methodology. Secondary outcomes include other measures of body composition (e.g., skeletal muscle mass/size, fat mass, muscle quality), physical function, muscle strength, power, and markers of muscle metabolism.	
Setting	There will be no restrictions by type of setting.	
Language	We will include articles reported in the English language. A list of possibly relevant titles where the full article is published in other languages will be provided.	

Abbreviations**:** GLP‐1RA, glucagon‐like peptide‐1 receptor agonists; LCD, low‐calorie diet; PUFA, polyunsaturated fatty acids; RCTs, randomized controlled trials; SGLT2i, sodium‐glucose co‐transporter 2 inhibitor.

### 2.2 Information Sources and Search Strategy

The International Clinical Trials Registry Platform Search Portal and ClinicalTrials.gov will be searched for ongoing or recently completed trials. PROSPERO will be searched for ongoing or recently completed systematic reviews.

Literature search strategies will be developed using medical subject headings (MeSH) and text words related to nutritional supplementation, weight loss, and body composition. Literature searches will be conducted in the following databases from inception: (1) MEDLINE, (2) Embase, and (3) Web of Science. Grey literature will be searched using Google Scholar (the first 300 search returns will be eligible for screening [14]) and OpenGrey. Combining search results from the four included databases (Embase, MEDLINE, Web of Science, and Google Scholar) is estimated to yield 98.3% of potential references [15]. Database searches will be supplemented with forward and backward citation tracking from included studies.

A draft MEDLINE search strategy is presented in Supplementary Material S2. Once finalized, the MEDLINE strategy will be adapted to the syntax and subject headings of the remaining databases. Search terms are broadly related to body composition, physical function, weight loss interventions, and supplementation. Preliminary searches and scanning of results from both MEDLINE and Web of Science returned several studies likely to be eligible upon full screening (“seed papers”; Supporting Material S3).

### 2.3 Selection Process

Literature search results will be uploaded to EndNote (v20 Clarivate). After the removal of duplicate titles, results will be transferred to Rayyan (https://www.rayyan.ai/) to facilitate a collaborative study selection process between reviewers. A calibration exercise will be undertaken to ensure members of the review team understand the eligibility criteria.

The review authors will independently screen the titles and abstracts yielded by the search for inclusion. For brevity, reasons for excluding trials at this stage will not be recorded. Texts will be obtained for all references not definitively excluded during title and abstract screening, for full‐text screening by unblinded review author pairs. We will resolve disagreements through discussion, and seeking additional information from study authors where necessary to confirm eligibility. Reasons for excluding trials will be recorded at this stage.

### 2.4 Data Collection

A data extraction form will be developed and piloted before full‐text data extraction. Data extraction will be completed by a single reviewer per study, and verified by a second reviewer. Disagreements will be resolved by discussion. We will contact the study authors to resolve any uncertainties.

### 2.5 Data Items

The extracted data will include but is not limited to: study details (author, year of publication, country of study), participant characteristics (health conditions, age, sex, ethnicity/race, baseline body weight, magnitude of weight loss achieved), intervention characteristics (supplement name, dose, frequency), and outcome measures (detailed below).

### 2.6 Outcomes and Prioritization

#### 2.6.1 Primary Outcomes

The primary outcome will be lean mass (either total, regional, or appendicular) expressed as absolute (e.g., kg) or relative (e.g., kg/height^2^) values.

#### 2.6.2 Secondary Outcomes

1. Skeletal muscle mass or size (either total, regional, or appendicular) expressed as absolute (e.g., kg, cm^2^) or relative (e.g., kg/height^2^) values.
2. Fat mass or size (either total, regional, or appendicular) expressed as absolute (e.g., kg, cm^2^) or relative (e.g., kg/height^2^) values.
3. Fat‐free mass or size (either total, regional, or appendicular) expressed as absolute (e.g., kg, cm^2^) or relative (e.g., kg/height^2^) values.
4. Body mass expressed as absolute (e.g., kg) or relative (e.g., kg/height^2^) values.
5. Measures of muscle “quality” expressed as muscle texture (e.g., echo intensity) or architecture (e.g., fiber orientation).
6. Physical function (e.g., sit‐to‐stand test performance, gait speed).
7. Strength (e.g., handgrip strength).
8. Muscle power (e.g., torque).
9. Measures of skeletal muscle metabolism (e.g., mitochondria function, phosphocreatine (PCr) depletion, protein synthesis).

### 2.7 Risk of Bias in Individual Studies

Two reviewers will independently assess the risk of bias using the United States National Heart Lung and Blood Institute (US NHLBI) quality assessment tool for controlled intervention studies. Disagreements will be resolved first by discussion and then by consulting a third author for arbitration.

### 2.8 Data Synthesis

A systematic narrative synthesis will summarize the characteristics and findings of the included studies. The narrative synthesis will explore the relationship and findings within and between the included studies, in line with guidance from the Centre for Reviews and Dissemination.

If study designs and variables are sufficiently homogeneous, we will conduct meta‐analyses using a random effects model. Continuous outcomes will be analyzed using weighted mean differences with 95% confidence intervals (CI), or using standardized mean differences (95% CI) if different measurement scales are used. Skewed and/or nonquantitative data will be presented descriptively. Where a study has more than two treatment groups, all eligible treatment arms (as determined using the above inclusion criteria) will be presented. We will follow conventional recommendations to include multiple groups in the meta‐analysis. We will attempt to contact the original authors of the study to obtain any relevant missing data. Where possible, data presented in figures only will be extracted using digital image analysis software (e.g., WebPlotDigitizer).

Statistical heterogeneity will be tested using the chi^2^ test (significance level: 0.1) and *I* ^2^ statistic (0%–40%: might not be important; 30%–60%: may represent moderate heterogeneity; 50%–90%: may represent substantial heterogeneity; 75%–100%: considerable heterogeneity). If high levels of heterogeneity among the trials exist (*I* ^2^ ≥ 50% or *p* < 0.1), the study design and characteristics in the included studies will be analyzed [16]. Potential sources of heterogeneity will be explored using subgroup (based on supplement type or comorbidity) or sensitivity (based on risk of bias assessment) analyses. Outcomes will be combined and calculated using statistical software (RevMan 5.1), according to the statistical guidelines referenced in the Cochrane Handbook for Systematic Reviews of Interventions (version 6.4, 2023). The Mantel–Haenszel method will be used for the fixed effect model where tests of heterogeneity are nonsignificant. If statistical heterogeneity is observed, the random effects model will be chosen. In the occurrence of substantial heterogeneity, a narrative, qualitative summary will be presented without quantitative analysis. For pooling of data from appropriate cross‐over studies, data must be presented using paired analyses [17].

### 2.9 Confidence in the Cumulative Estimate

The quality of evidence for all outcomes will be judged using the Grading of Recommendations Assessment, Development and Evaluation working group methodology. The quality of evidence will be assessed across the domains of risk of bias, consistency, directness, precision, and publication bias. Additional domains may be considered where appropriate. Quality will be adjudicated as high (further research is very unlikely to change our confidence in the estimate of effect), moderate (further research is likely to have an important impact on our confidence in the estimate of effect and may change the estimate), low (further research is very likely to have an important impact on our confidence in the estimate of effect and is likely to change the estimate), or very low (very uncertain about the estimate of effect).

## 3 Discussion

The primary treatment aim for people with obesity is sustained long‐term weight loss. Regardless of the treatment approach to weight loss (lifestyle, pharmacological, and/or surgical), successful interventions can be expected to improve cardiometabolic health markers in their participants [2, 18]. However, reductions in lean mass that often occur concomitantly with weight loss may attenuate any associated health benefits [4] and reduce physical function. Importantly, low lean mass and/or poor physical function are associated with loss of functional independence [19], hospitalization, and nursing home admission [20]. Preservation of lean mass during weight loss requires the presence of adequate anabolic stimuli. This could include targeted nutritional supplements [12]. However, the role of nutritional supplements in lean mass accretion during weight loss is not fully understood. This review and meta‐analysis will systematically summarize the evidence to date. We primarily aim to quantify the magnitude and direction of change in lean mass, with or without nutritional supplementation, following intentional weight loss. We will also provide a narrative synthesis and methodological quality assessment of the published literature, and identify knowledge gaps to be addressed in future research. The findings of this review will be published in peer‐reviewed journals and will inform future research designed to preserve lean mass in people with obesity undergoing weight loss.

## Author Contributions

**Emily James:** conceptualization, methodology, writing–original draft, writing–review and editing. **Ryan J. Kelsey:** conceptualization, methodology, writing–review and editing. **Jack A. Sargeant:** conceptualization, methodology, writing–review and editing. **Joe Henson:** conceptualization, methodology, writing–review and editing. **Tom Yates:** conceptualization, methodology, writing–review and editing. **Thomas J. Wilkinson:** conceptualization, methodology, writing–original draft, writing–review and editing. All authors have read and approved the final version of the manuscript.

## Conflicts of Interest

The authors declare no conflicts of interest.

## Transparency Statement

The lead author Emily James affirms that this manuscript is an honest, accurate, and transparent account of the study being reported; that no important aspects of the study have been omitted; and that any discrepancies from the study as planned (and, if relevant, registered) have been explained.

## Supporting information

Supporting information.

## Data Availability

Data sharing is not applicable to this article as no new data were created or analyzed in this study. Data will be made available upon reasonable request. Further enquiries can be directed to the corresponding author. T.J.W. has full access to all of the data in this study and takes complete responsibility for the integrity of the data and the accuracy of the data analysis.
